# RNA Profiles of Porcine Embryos during Genome Activation Reveal Complex Metabolic Switch Sensitive to *In Vitro* Conditions

**DOI:** 10.1371/journal.pone.0061547

**Published:** 2013-04-29

**Authors:** Olga Østrup, Gayla Olbricht, Esben Østrup, Poul Hyttel, Philippe Collas, Ryan Cabot

**Affiliations:** 1 Institute for Basic Medical Sciences, Faculty of Medicine, University of Oslo and Norwegian Center for Stem Cell Research, Oslo, Norway; 2 Department of Clinical Veterinary and Animal Sciences, Faculty of Health and Medical Sciences, University of Copenhagen, Frederiksberg C, Denmark; 3 Department of Mathematics and Statistics, Missouri University of Science and Technology, Rolla, Missouri, United States of America; 4 Institute of Clinical Dentistry, University of Oslo, Oslo, Norway; 5 Norwegian Center for Stem Cell Research and Institute of Immunology, Oslo University Hospital, Oslo, Norway; 6 Department of Animal Sciences, College of Veterinary Medicine, Purdue University, West Lafayette, Indiana, United States of America; The Babraham Institute, United Kingdom

## Abstract

Fertilization is followed by complex changes in cytoplasmic composition and extensive chromatin reprogramming which results in the abundant activation of totipotent embryonic genome at embryonic genome activation (EGA). While chromatin reprogramming has been widely studied in several species, only a handful of reports characterize changing transcriptome profiles and resulting metabolic changes in cleavage stage embryos. The aims of the current study were to investigate RNA profiles of *in vivo* developed (ivv) and *in vitro* produced (ivt) porcine embryos before (2-cell stage) and after (late 4-cell stage) EGA and determine major metabolic changes that regulate totipotency. The period before EGA was dominated by transcripts responsible for cell cycle regulation, mitosis, RNA translation and processing (including ribosomal machinery), protein catabolism, and chromatin remodelling. Following EGA an increase in the abundance of transcripts involved in transcription, translation, DNA metabolism, histone and chromatin modification, as well as protein catabolism was detected. The further analysis of members of overlapping GO terms revealed that despite that comparable cellular processes are taking place before and after EGA (RNA splicing, protein catabolism), different metabolic pathways are involved. This strongly suggests that a complex metabolic switch accompanies EGA. *In vitro* conditions significantly altered RNA profiles before EGA, and the character of these changes indicates that they originate from oocyte and are imposed either before oocyte aspiration or during *in vitro* maturation. IVT embryos have altered content of apoptotic factors, cell cycle regulation factors and spindle components, and transcription factors, which all may contribute to reduced developmental competence of embryos produced *in vitro*. Overall, our data are in good accordance with previously published, genome-wide profiling data in other species. Moreover, comparison with mouse and human embryos showed striking overlap in functional annotation of transcripts during the EGA, suggesting conserved basic mechanisms regulating establishment of totipotency in mammalian development.

## Introduction

Initial embryonic development is accompanied by complex genomic and metabolic changes that occur at different levels in a highly coordinated manner. The maternal and paternal genomes are decondensed and epigenetically remodelled to totipotency shortly after fertilization [1]. In a species-specific and stage-specific manner, the newly formed diploid embryonic genome becomes activated and embryo-specific transcripts become transcribed. The initiation of mRNA synthesis in the cleavage stage embryo is referred to as embryonic genome activation (EGA) and occurs during the 4-cell stage in the porcine embryo [2]. EGA promotes a dramatic reprogramming of gene expression coupled with the generation of novel transcripts that are not expressed in oocyte. Hereby, the developmental program governed by maternal transcripts/proteins is dramatically switched to that dominated by transcripts/proteins from the newly formed embryonic genome. While EGA can be exactly timed by appearance of active transcription, gradual degradation of maternal transcripts and their replacement by embryonic transcripts is stretched over several cell divisions and this process is often referred to as the maternal-to-embryonic transition [3]. Because the initiation of EGA occurs within a period of presumably few hours, we expect that the period immediately preceding and immediately following EGA will be characterized by marked differences in transcripts found in the embryo.

Prior to EGA, the embryonic cytoplasm consists mostly of transcripts produced and stored during oocyte maturation. In addition, recent data indicate that spermatozoa may also provide the zygote with a unique set of paternal mRNAs that contribute to fine tuning of reprogramming before the EGA onset [4]. However, the exact role of paternal mRNAs, as well as identification of specific key regulators still remains to be clarified. Following EGA, transcripts required for oocyte maturation and fertilization are no longer needed and undergo gradual degradation. The remaining transcripts are responsible for development immediately preceding EGA and drive of initial embryonic development. These transcripts are responsible for completing meiosis in the oocyte, terminating the developmental program of both gametes, avoiding apoptosis, initiating mitosis, reorganizing chromatin, and preparing for the first cellular differentiation event in the embryo; formation of the trophectoderm and inner cell mass populations. EGA thus represents a breaking point for establishment of totipotency. From a transcriptome point of view, EGA onset is reflected by massive usage of transcripts required for initiation of transcription and by degradation of transcripts that prevent precocious transcription initiation. However to our knowledge, cytoplasmic processes accompanying EGA have not been addressed on transcriptomic level so far. The characterization of these would significantly advance our understanding of supporting mechanisms enabling proper activation and regulation of reprogrammed genome in variety of biological processes.

Advances in genome-wide transcriptome profiling, accompanied by both functional analysis and reference data mining, provide a unique set of tools for the identification of signalling pathways that may serve critical regulatory roles in the processes that lead to cellular reprogramming towards totipotent and pluripotent states. Genome-wide analysis has been reported in mouse, zebrafish, *Drosophila*, bovine and human embryos surrounding the respective species-specific time of EGA using various approaches [5]–[10]. However, despite its importance as an animal model, transcriptome analysis of the pig embryo during this developmental window remains to be investigated. Moreover, identification of the differences between the transcriptomes found in porcine embryos produced *in vitro* and such developed *in vivo* may provide valuable insight into the mechanisms that lead to the reduced developmental potential observed in *in vitro* produced porcine embryos and thereby provide opportunities to develop improved conditions for handling embryos in the laboratory.

## Materials and Methods

### Embryo Collection and Culture

#### *In vivo* (IV) embryo collection

Sows were slaughtered at defined time points after insemination and the uteri were transported to the laboratory at 37°C within 1 h. Porcine uteri were donated by a local abattoir (Kosakgaarden, Slangerup) to use to meet the research objectives set forth in this manuscript. The uterine horns were flushed with pre-warmed phosphate buffer saline (PBS) enriched with 1% foetal bovine serum (FBS) to collect the embryos. The embryos were recovered from the flushing medium at Day 2 (2-cell stage) and Day 3 (4-cell stages).

#### *In vitro* (IVF) embryo production

Prepubertal gilt ovaries were obtained from a local slaughterhouse; cumulus-oocyte complexes (COCs) were aspirated from antral follicles with an 18-gauge needle and a disposable 10 ml syringe. COCs were resuspended in HEPES-buffered medium containing 0.01% polyvinyl alcohol (PVA) [11]. COCs were matured *in vitro* as described previously [11]. Briefly, groups of 50–70 COCs were placed in 500 µl of maturation medium composed of Tissue Culture Medium 199 (TCM-199; Gibco BRL, Grand Island, NY) supplemented with 0.14% PVA, 10 ng/ml epidermal growth factor, 0.57 mM cysteine, 0.5 IU/ml porcine FSH, and 0.5 IU/ml ovine LH for 42–44 hours at 39°C in a humidified atmosphere of 5% CO_2_ in air. Following maturation, COCs were vortexed in 0.1% hyaluronidase in HEPES-buffered medium containing 0.01% PVA for 4 minutes to remove the cumulus cells. Mature denuded oocytes were then placed in a modified Tris-buffered medium (mTBM) and fertilized according to an established protocol [12], using fresh, extended boar semen. Briefly, boar semen was extended in Modena Boar Semen Extender (Swine Genetics International, Cambridge, IA) and kept at 17.5°C for up to three days. Before fertilization, one milliliter of extended semen was mixed with 10 ml of Dulbecco’s Phosphate Buffered Saline and centrifuged at 1000×g, 25°C, for four minutes. Washing was repeated three times; following the third wash, the sperm pellet was resuspended in mTBM. The final concentration of sperm used in the fertilization experiments was 5×10^5^ cells/ml. Groups of 30–35 oocytes were placed in 100 µl of mTBM; gametes were co-incubated five hours at 39°C and 5% CO_2_. Following gamete co-incubation, presumptive zygotes were placed in PZM3 containing 3 mg/ml fatty acid free BSA [13] for embryo culture. Embryos were maintained at 39°C, 5% CO_2_ and 100% humidity until the appropriate cell stage. Two-cell stage embryos were collected 24 hours after gamete mixing; four-cell stage embryos were collected 48 hours after gamete mixing. In both cases, only embryos that could be identified morphologically as 2-cell or 4-cell stage embryos at the above-specified time points were used. Developmental competence is stated in Table 1. All experiments involving porcine oocytes and in vitro produced porcine embryos were performed with the specific approval of the Purdue Animal Care and Use Committee. Porcine ovaries were donated by a local abattoir (Indiana Packers) to use to meet the research objectives set forth in this manuscript.

**Table 1:** Developmental competence of *in vitro* produced embryos.

Replication	No. of oocytes cultured	Cleavage rate		
*(Date of IVF)*		*2-cell* 1	*4-cell* 2	*Blastocyst* 3
1 (5/24)	450	50/175 (29%)	65/175 (37%)	12/100 (12%)
2 (5/25)	430	46/170 (27%)	69/170 (41%)	12/90 (13%)
3 (5/31)	460	52/180 (29%)	73/180 (41%)	10/100 (10%)
4 (6/1)	410	41/160 (26%)	68/160 (43%)	8/90 (9%)

Developmental competence of *in vitro* produced (IVT) embryos from 4 biological replicates stated as a percentage of cleavage rates at 2- cell, 4-cell, and blastocyst stage.

1 2-cell stage embryos collected 30 hours after IVF.

2 4-cell stage embryos collected 48 hours after IVF.

3 Blastocyst stage embryos collected 6 days after IVF.

### RNA Extraction

Total RNA was isolated from pools of *in vivo* and *in vitro* produced porcine embryos at 2- and 4-cell stage using the RNeasy Micro kit (Qiagen, #74004) according to the manufacturer instructions. For the 2-cell stage, one biological replicate was collected from *in vivo* embryos (pool of 20 embryos) and two biological replicates (each pool of 40 embryos) were collected from *in vitro* embryos. For the 4-cell stage, two biological replicates were from *in vivo* embryos (each pool of 20 embryos) and one biological replicate was obtained from *in vitro* embryos (pool of 80 embryos). Thus, a total of six samples were employed in the study. Total RNA was treated with RiboMinus™ (Invitrogen, K1550-01) according to the manufacturer’s instructions, to remove ribosomal RNA; non-amplified RNA was then submitted to the Genomics Core Facility at Purdue University for RNA sequencing.

### RNA Sequencing and Data Analysis

RNA deep sequencing was performed using the SOLiD system (Applied Biosystems). Sequencing data (deposited in NCBI Sequence Read Archive acc.nr. SRP018847) were analysed using Bioscope whole transcriptome software with paired-end data and aligned to the porcine genome sequence available at the time, SusSc 62 (160 507 CDS in annotation). Transcripts that mapped to annotated genes were retained for the analysis, leaving a total of 9191 annotated genes for differential expression analysis (also accounting for transcripts with differential presence). Recently, the swine genome was fully sequenced [14], however due to the persisting limitation in use of porcine data in freely available bioinformatics tools used in the current study, the new annotation did not significantly influence the output of the present analysis.

Sequencing by SOLiD resulted in two read lengths; F3 with 50 bp and F5 with 35 bp. The details of the RNA sequencing results are listed in Table 2. In general, all six samples represented a low percentage of mappable Tags varying from 7% (4-cell stage, *in vivo*, replicate 2; 4 cell IVV2) to 30% (2-cell stage, *in vitro*, replicate 2; 2 cell IVT2). However, considering the very low input cell numbers and annotation restrictions of porcine genome [15]–[17], these results were considered as reliable and subjected for further statistical analysis and RT-qPCR validation. The correlation coefficients between equivalent biological replicates were 0.91 (2-cell stage) and 0.92 (4-cell stage).

**Table 2:** Output of RNA sequencing.

Group	Total tags	Mappable tags/%	# CDS in annotation	CDSes with tags	Tags matching CDSes	# exons in annotation	Exons with tags
2 cell IVV	17 372 244	2320994/**13**	160507	1983	107498	168998	2668
2 cell IVT1	16 861 264	1556761/**9**	160507	925	34928	168998	1323
2 cell IVT2	41 729 110	12791613/**30**	160507	15076	984448	168998	18283
4 cell IVV1	28 501 060	2774378/**9**	160507	2232	28183	168998	3260
4 cell IVV2	22 521 782	1682540/**7**	160507	1938	28777	168998	2781
4 cell IVT	8 968 590	2220189/**24**	160507	3142	324952	168998	4510

Quality control statistics of RNA sequencing by SOLiD system. In total, 20 *in vivo* developed (IVV) embryos were used per sample. From *in vitro* produced (IVT) embryos, 40 embryos per sample at 2-cell stage and 80 embryos per sample at 4-cell stage were pooled.

IVV *in vivo* developed embryos; 20 embryos per sample.

IVT*in vitro* produced embryos; 40 (2-cell stage) and 80 (4-cell stage) embryos per sample.

It is important to note that this experimental design does not allow for detection of the contribution of paternal RNAs. All embryos used in this study were monospermic and thus the amount of paternal RNA does not exceed 50 fg per embryo [18]. The RNA was not amplified prior to sequencing and as recently shown; two rounds of amplification are required for sequencing of RNA isolated from 20 mil sperms [19], [20].

### RT-qPCR Validation

RNA from GV oocytes (3 pools of 500), 2-cell and 4-cell stage embryos, and blastocysts was isolated from embryos in three biological replicates (Table 1, three first rows) using Qiagen RNeasy MicroKit (Qiagen, # 74 004) according to the manufactures instruction applying 75 uL (embryos) or 350 uL (GV oocytes) RLT buffer for lysis, adding RNA carrier and omitting mechanical homogenization (expect GV oocytes). A total of 0.25 ng/ml of control RNA kanamycin (Promega #C1381) was added to RLT buffer prior to the RNA extraction and used as the reference control by qPCR. RNA was reverse transcribed by the iScript Select cDNA synthesis Kit (BioRad #170–8896) and random hexamer priming according to the manufactures instructions.

Primers were designed for 5 transcripts enriched at the 2-cell stage (*SMARCA5, NUP133, USP7, UBE2Q1,* and *THOC2*) and for 7 transcripts enriched at 4-cell stage (*ELL2, TERF2, TIP48 (RuvB), RNF20, ZNF575, SNURP,* and *THOC3*). Sequences, annealing temperatures and fragment sizes are shown in Table 3. Primer efficiencies for all primer pairs were determined by performing PCR on serially diluted templates to generate standard curves (R values above 0.98; E values 85–100%). Dynamics of the transcripts were confirmed by quantitative (q)PCR. qPCR was performed with the iCycler MyiQ real time PCR detection system and SYBR Green (BioRad). Primers pairs gave no signal in reactions lacking template (not shown). Relative expression was determined by the ΔΔC_T_ method using level of transcripts in blastocysts (*ZNF575*) or GV oocytes (others) as an input value. Fold changes between 4- and 2-cell stages determined by qPCR were compared to fold changes identified by RNA-seq.

**Table 3:** Overview of primer sequences used for validation of RNA sequencing.

		qPCR primers		
Gene		Sequence 5-3	Annealing t	Fragment size
SMARCA5	F	ACCTGATGGCAGAGGAAGAA	59,8	
	R	TCAGCACAGCTGTTGCATTT	60,61	220 bp
NUP133	F	TATCCCATGGACAGCAACAA	59,92	
	R	CAGCTGCTCTGTCACCATGT	60,05	130 bp
USP7	F	CCCTTGATGAGCTGATGGAT	60,03	
	R	AAATCACATCGACACGGTGA	59,97	126 bp
UBE2Q1	F	CACTGCAACATCACGGAATC	60,12	
	R	ACACAGGTCGGAGATGATCC	59,93	159 bp
THOC2	F	CGCTTGGATCCAGAAACATT	60,07	
	R	CAGCAATCAGCTTGGCATAA	59,98	157 bp
ELL2	F	GTTTCAGATGCAGTGCCTGA	59,9	
	R	ATATGGCCGCTGAGAAACAC	60,1	102 bp
TERF2	F	GGAGTGCATTTGTTCCAGGT	59,97	
	R	CCAAGGCAGTCAGGTCTAGC	60,01	164 bp
TIP48 (RuvB)	F	ATCGGACTGGAGACCTTCCT	60,07	
	R	GGCATCCTGGTACTCCTTCA	60,07	171 bp
RNF20	F	AGAAGCCCAGTCTGACCTGA	59,99	
	R	GTCTTCGAGGTAGCGGACTG	60,01	150 bp
ZNF575	F	TTGAGTCGGGAGTCCATCTC	60,2	
	R	AAGCTAACACGGGGAAACCT	60,00	175 bp
SNURP	F	CTACAGCCCTGGAAGCACTC	60,01	
	R	TCAGAGGCCTTGTGTGTGAG	60,02	195 bp
THOC3	F	GGAAGCTGGAACTGTGAAGC	60	
	R	AGCTCCAAGAGGACAGACCA	59,99	127 bp

### Gene Ontology (GO) Classification and KEGG Pathways

To determine the main biological processes involved in nuclear and cytoplasmic remodelling of embryos for embryonic genome activation, the differentially expressed gene lists were uploaded into DAVID (database for annotation, visualization and integrated discovery; [21]. The *homo sapiens* genome was used as the background gene list using e!Ensemble. Only orthologes with above 50% identity were used, and all genes below 75% identity were individually confirmed by alignment in BLAST. The enriched functional annotation terms are identified and listed according to their enrichment P-value and fold enrichment score by DAVID. The pathways containing the differentially expressed genes were identified by KEGG (The Kyoto Encyclopaedia of Genes and Genomes) (http://david.abcc.ncifcrf.gov).

### Clustering of Differentially Expressed Genes

A heatmap of differentially expressed genes was made to visualize similarities in gene expression between samples. Expression values of differentially expressed genes were Log2 transformed and reordered according to hierarchical clustering using the “stats” package for R.

## Results and Discussion

Much of the data published regarding transcript abundance in the porcine embryo are based on the analysis of candidate genes, which are generally selected for analysis due to their roles in pathways hypothesized to be important during this stage in development. While such experimentally derived data are critical to test hypotheses related to the functional roles of specific pathways during early development, these data provide only very narrow characterization of processes accompanying EGA. Moreover, insufficient *in vitro* conditions are an enormous obstacle that limit our ability to efficiently produce developmentally competent porcine embryos in the laboratory; large proportion of in vitro produced porcine embryos fail to develop beyond the stage of embryonic genome activation. This limitation hinders our efforts to use the pig model in biomedical research. Understanding transcriptional network regulation surrounding the time of EGA will enable us to identify the pathways that contribute to totipotency and may provide insight into the reasons behind the developmental compromise of in vitro produced embryos. In this series of experiments we were interested in determining the changes in RNA profiles in porcine embryos before and after EGA. To this end, we collected RNA from 2-cell and 4-cell stages, and analysed the transcriptome by RNAseq using the SOLiD system.

### Statistical Analysis

Due to costly resources of porcine *in vivo* developed embryos and high variability between *in vitro* produced embryos, the amount and quality of porcine embryos is one of the major limiting factors for genome-wide studies. Therefore, considering the low percentage of mappable reads it was crucial to establish the proper statistical model in order to achieve reliable and reproducible data.

The expression counts for each gene were normalized by the total number of reads per sample via a Poisson rate model with cell stage (2-cell; 4-cell) and embryo source (*in vitro; in vivo*) effects. This type of normalization and modelling has been effectively used in many RNA-seq applications [22], [23]. To allow for more flexibility in the modelling, both a standard Poisson model and a Poisson model that allows for overdispersion were fit for each of the genes and results compared for the hypotheses tests of interest (Table S1). The Poisson model that allows for overdispersion accounts for the replicated nature of the data and accommodates within-group heterogeneity, but is more conservative than the standard Poisson model [23]. Genes with expected cell counts for the full model that are larger than or equal to 5 in at least two samples were retained for the analysis, and those not meeting this criterion were excluded in order to maintain the asymptotic behaviour of the likelihood ratio test. This left a total of 2596 genes for the analysis. To correct for multiple testing, the False Discovery Rate (FDR) was controlled using the Benjamini-Hochberg (BH) method at 5%. Thus, genes with Benjamini-Hochberg adjusted p-values less than 0.05 are identified as differentially expressed.

The standard Poisson model identified 2101 genes (80.9%) with significant differential expression between the 2-cell stage and late 4-cell stage, while the Poisson model with overdispersion identified 1112 significant differentially expressed genes (42.8%) between the two developmental stages. Note that the majority of the significant genes that are identified with the overdispersed Poisson model are also significant in the standard Poisson model (1091/1112), illustrating the conservative nature of the overdispersed Poisson model. The gene list for both models with statistical data and log fold changes between 2- and 4-cell stages can be found in Table S1. Due to the low cell number input and relatively low RNA sequencing output, further analysis was performed only on genes found as differentially expressed by the Poisson model with overdispersion.

The differentially expressed genes could be divided into 4 basic groups: a group of maternal transcripts present at the 2-cell stage, but absent at 4-cell stage (n = 127; 2-cell only); a group of maternal-embryonic transcripts highly abundant at 2-cell stage, but less abundant at the 4-cell stage (n = 519; 2-cell enriched); a group of maternal-embryonic transcripts with low abundance at the 2-cell stage, but with high abundance at the 4-cell stage (n = 332; 4-cell enriched); and a group of embryonic transcripts only found at 4-cell stage (n = 19; 4-cell only). Similar categories of transcripts were identified in previous studies [5], [6], [8].

### Validation of RNA-seq Data

**Figure 1:**
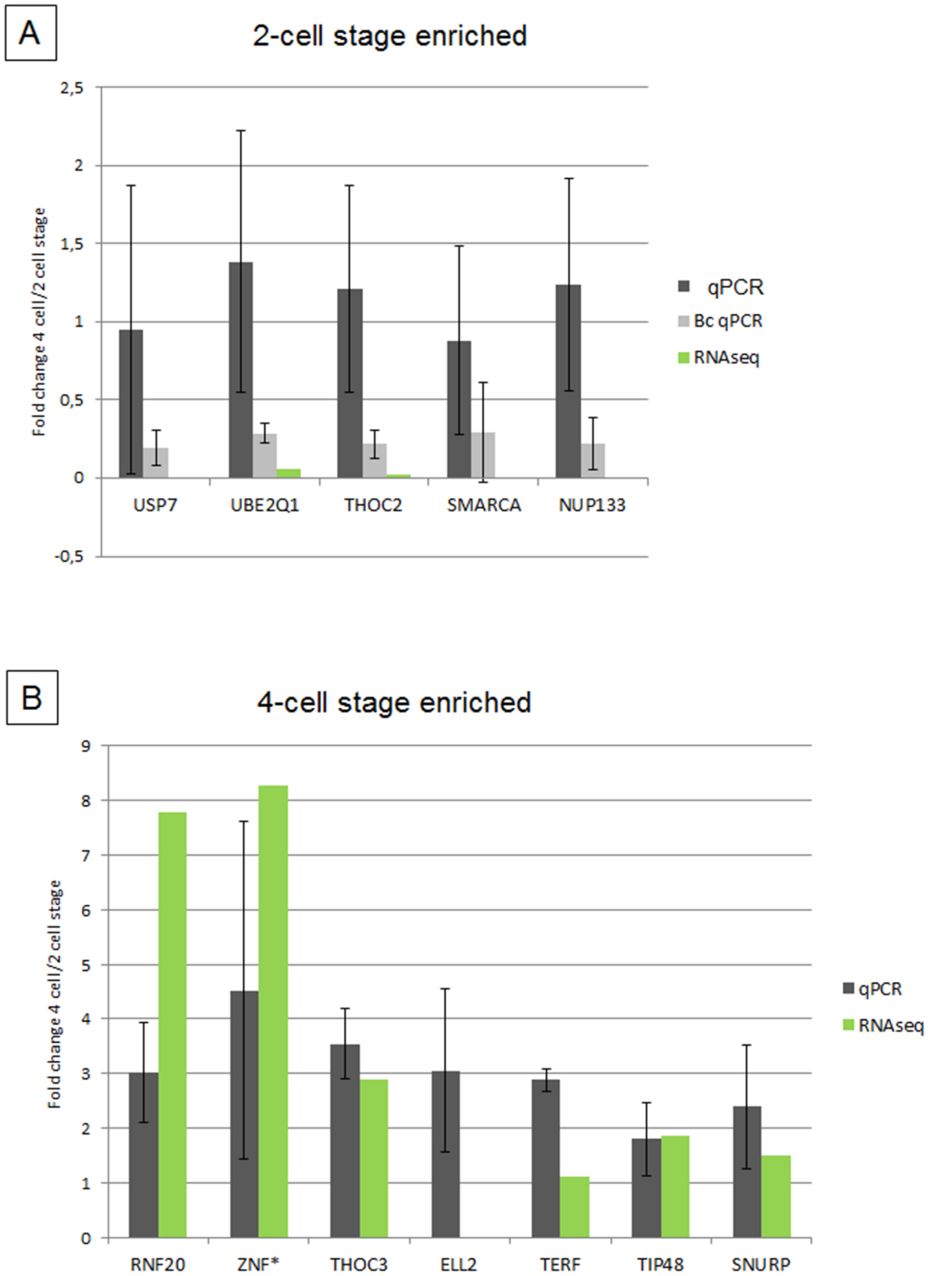
Validation of RNA sequencing by RT-qPCR. Graphs showing fold changes between 4-cell/2-cell stages derived from ddCt values (black bars; qPCR) and identified by RNA sequencing (green bars; RNA-seq). Figure 1A shows the validation results for genes identified as enriched at 2-cell stage. Grey bars named Bc qPCR show fold changes between blastocyst/2-cell stages and demonstrate the tendency towards continuous decrease in the amount of these transcripts. Figure 1B shows the validation results for genes identified as enriched at 4-cell stage.

For validation of RNA-seq data, 5 two-cell enriched or 2-cell only transcripts (*USP7, UBE2Q1, THOC2, SMARCA5, NUP133*) and 7 four-cell enriched transcripts (*RNF20, ZNF575, THOC3, ELL2, TERF, TIP48, SNURP*) were selected for analysis by RT-qPCR randomly or by being genes of interests in sections below. While transcripts that increased in abundance (from the 2-cell to the 4-cell stage) displayed a very strong correlation with RNA-seq values (Figure 1B), the transcripts that decreased in abundance (from the 2-cell to the 4-cell stage) did not show any significant changes (Figure 1A). However, significant decreases in the levels of these transcripts were observed when compared to the blastocyst stage, indicating their gradual degradation.

Lack of correlation between RNA-sequencing and qRT-PCR validation in the pre-EGA enriched transcripts have been also reported in other studies, however the reasons behind these observations were not further investigated [6], [16], [17], [24]. We hypothesize that the methodological differences between the preparation of the samples for RNA-seq and RT-qPCR together with the molecular characteristics of maternally inherited transcripts at the time of the EGA may explain the observed discrepancy. Maternal transcripts present in the 2-cell stage embryos become destabilized at the 4-cell stage and are prone to massive usage or degradation by different mechanisms [3]. These destabilized transcripts are rendered sensitive to degradation during the RNA-seq workflow and are protected prior to the RT in the RT-qPCR replicates. Therefore, RNA-seq may not effectively detect these transcripts, or detect them at only a very low level at the 4-cell stage. As a result, such transcripts appear enriched at the 2-cell stage. Therefore, the specific features of maternally inherited transcripts and their behaviour during the maternal-to-embryonic transition have to be considered in the sequencing studies.

However, the presented RNA-seq data clearly show that both 2-cell stage only and 2-cell stage enriched transcripts are maternally inherited and undergo gradual degradation towards the blastocyst stage. Abundance of 4-cell stage enriched transcripts increases towards the 4-cell stage and shows similar correlations as RNA-seq experiments performed on transcriptionally active cells.

### Specific Features of Transcriptome during EGA

**Figure 2:**
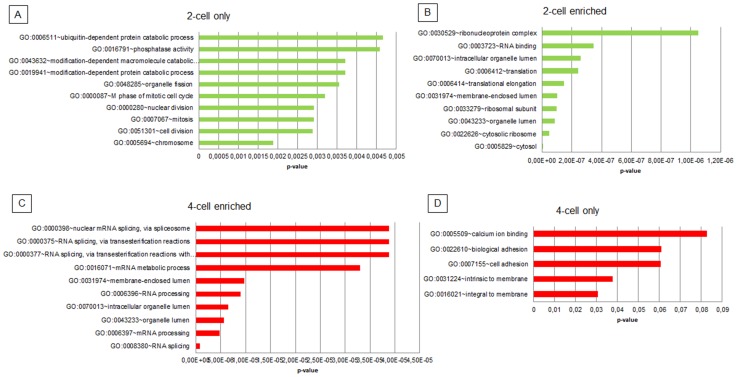
Top 10 significantly enriched GO terms. Graphs showing the top ten significantly enriched GO terms (y-axis) and their p-values (x-axis) of differentially expressed genes found only at the 2-cell stage (2A), enriched at the 2-cell stage (2B), enriched at the 4-cell stage (2C) and found only at the 4-cell stage (2D).

In order to identify functional groups of genes and transcripts of interest, gene lists from all 4 groups were analysed by GO terms (Table S2 A-D) and subsequent clustering of significantly enriched GO terms in DAVID (Table S3 A–D). Table 4 provides an overview of the number of genes, identified GO terms, and GO term clusters. The 10 most significantly enriched GO terms from each group are shown in Figure 2.

**Table 4:** Overview of the numbers of genes, their human orthologes, significantly enriched GO terms and GO term clusters identified by comparison of differential expression between the 2-cell and the 4-cell stage.

Group	Differentially present transcripts N	N with human orthologes	Significantly enriched GO terms	Number of GO term clusters	Identified KEGG pathways
2-cell only	127	119	44	19	1
2-cell enriched	519	497	195	122	5
4-cell enriched	332	287	98	60	6
4-cell only	19	15	10	0	0

#### 2-cell only

The most significantly enriched GO terms associated with transcripts found in 2-cell stage embryos only included *chromosome*, *cell division*, *mitosis*, *nuclear division*, *M-phase*, and *organelle fission* (Figure 2A). This is in accordance with the concept of transcription-independent, maternally driven regulation of the first cell divisions, and thus need for cell cycle control over several days during the initial mammalian development. According to the GO terms, KEGG pathway analysis identified only one pathway, *Cell Cycle*, as significantly enriched. Among the top-scored GO terms were also protein catabolic processes, proteolysis, and ubiquitin-dependent protein catabolic processes. These GO terms also formed top-scored cluster and clearly reflect massive degradation of maternal proteins during the phase of maternal-to-embryonic transition. Moreover, proteolysis is also an essential step in protein processing and may lead to formation of functional proteins enabling continuation of metabolic processes and preparation for EGA [25], [26]. Interestingly, we also found GO terms including chromatin remodelling complex (*APPL2, SMARCA5, MYSM1* and *TBL1XR1*) and chromosome organization (e.g., *SETD1A, LATS1*), both of which are involved in chromatin changes during cell cycle [27]–[29] and also participate in remodelling of maternal and paternal chromatin after fertilization [30], [31].

#### 2-cell enriched

The 2-cell only transcripts could be clearly related to degradation of maternal proteins and the extensive stock-pile of factors required for cell cycle regulation. Similarly, maternal-embryonic, 2-cell enriched transcripts demonstrated similar events. The top scored GO terms were *cytosol* and *ribosomal subunits*; these components are known to be of maternal origin, stored in high amounts and required for survival of embryos prior to EGA [2], [32]. In agreement with capacious storage of ribosome machinery, the transcripts involved in downstream events of RNA processing such as *RNA-binding*, *translation*, and *translation elongation* were found in significantly enriched GO term clusters, indicating massive synthesis of functional proteins from maternally stored RNAs (Figure 2B).

Noticeably, GO terms including *organelles*, *nucleoplasm*, and *cytoskeleton* were also in the two top scored clusters. Moreover, more than 21% of the 2-cell enriched transcripts were contained in GO:0043228, *non-membrane bound organelle*, emphasizing that ooplasm provides not only the regulators, but also the important components of the machinery driving early embryonic development. The transcripts included in this GO term could also be directly linked to chromatin (e.g. *H2AZ, H3.3B,* and *MBD3*) and chromatin regulation (e.g., *FBOX5, SMARCA4, THAP7, hsp27, HDAC1,* polymerase II polypeptide F, and *p53*). KEGG pathway analysis identified 9 pathways, from which hsa05016: Huntingtons disease contained the most genes from the list (*calthrin, dynactin, dynein, HDACs, p53, CAPS3,* and *Cx*).

#### 4-cell enriched

In striking contrast to maternally-stored transcripts functioning in the pre-EGA period, transcripts increasing at the 4-cell stage (i.e., after EGA) strongly reflect the initiation of transcription and related processes. The top scored GO terms include *RNA splicing* and *(m)RNA processing* and *DNA metabolic processes* (Figure 2C). Accordingly, KEGG pathway analysis identified 6 pathways, from which “*Spliceosome*” was the most significant. Moreover, several novel GO terms appear as significantly enriched and correlate with chromatin modification and organization (Cluster7; *CENPF, RUVBL2,* and *NIMA*), histone acetylation (Cluster13; *HDAC2*), and transcription factors (Cluster22; *MEF2C, RBBP7, USF2,* and *MYC*). The continuous replacement of maternally derived factors by factors produced by the embryo is reflected by the abundance of transcripts related to protein-metabolic processes and protein transport (Clusters 4 and 5).

#### 4-cell only

Due to the low numbers of genes identified in this group, only two GO terms were found as significantly enriched; integral to membrane and intrinsic to membrane (Figure 2D). Eight genes could be assigned to GO-term cellular (biological) adhesion, which may reflect the preparation of embryos for early compaction at the morula stage as seen in bovine embryos [10].

### Complex Metabolic Switch Accompanies Transition through Permissive State

GO term analysis revealed that non-overlapping lists of 2-cell and 4-cell enriched genes could be grouped into functionally similar/identical GO terms and clusters. This indicates that similar processes are taking place before and after the EGA, although involving different transcripts. Two of the highly enriched GO terms overlapping between the groups were “*protein catabolic process*” and “*RNA splicing*” (Table S4).

#### Protein catabolic processes

**Figure 3:**
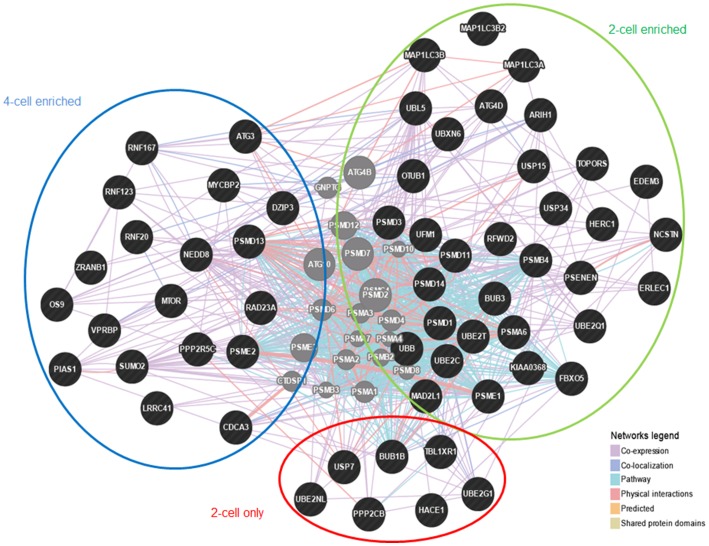
Gene network of differentially expressed genes annotated the GO term “Protein catabolic processes”. Gene network of differentially expressed genes annotated the GO term “Protein catabolic processes”, modified for better visualization of groups of genes found only at the 2-cell stage (red circle) and identified as enriched at the 2- (green circle) or the 4-cell stage (blue circle).

Transcripts involved in protein catabolic processes were highly abundant at both the 2- and 4-cell stages. Most of them could be directly linked to ubiquitin (*UBB*), whose transcripts are significantly enriched at the 2-cell stage. Ubiquitin function is linked to three types of enzymes. An ubiquitin-activating enzyme, E1, first activates ubiquitin by covalently attaching the molecule to its cysteine residue. Activated ubiquitin is then transferred to cysteine of E2 enzyme. Once conjugated to ubiquitin, the E2-UBB complex binds one of several ubiquitin ligases, or E3s, via a structurally conserved binding region. The E3 molecule is responsible for binding the target protein substrate and transferring the ubiquitin from the E2 cysteine to a lysine residue on the target protein [33]. A cell usually contains only a number of E1 molecules, a greater diversity of E2 molecules, and a very large variety of E3 molecules. Their inherent stability may be a reason why the E1 transcripts were not identified as significantly enriched in neither 2- nor 4-cell stages. Interestingly, the abundance of several E2 and E3 transcripts differed between the 2- and 4-cell stages. While E2 ubiquitin-conjugating enzymes (UBE2; G1, N, 2C, 2Q1, and 2T) appear enriched only at 2-cell stage, E3 ligases (RNF20, RNF123, RNF167, and MYCBP2) are mostly found in the group of the 4-cell enriched transcripts. This may indicate a time-dependent usage of ubiquitin-related machinery throughout the maternal-to-embryonic transition, that is, E2-UBB complexes preparation at 2-cell stage and their subsequent processing by E3 at 4-cell stage. Gene network analysis (www.genemania.com), however, did not reveal any direct pathway connections between E2 enzymes enriched at 2-cell stage and E3 enzymes enriched at 4-cell stage (Figure 3). This suggests the presence of several, independent protein catabolic processes occurring at different developmental time-points. The finding also corresponds to specific functions of the E2 and E3 enzymes. All E2 types identified here have been reported to be involved in DNA repair (*UBE2N* and *UBE2T*), cell cycle progression and regulation (*UBE2T, UBE2Q1,* and *UBE2C*), thus processes crucial for the pre-EGA period [34]–[36]. The identified RNF proteins on the other hand, are known to participate in transcription-related chromatin remodeling and organization [37], [38], which are processes characteristic for the post-EGA period.

Similar to the E2 enzymes, ubiquitin specific peptidases (USP) have also been found as significantly enriched only at the 2-cell stage. USPs cleave ubiquitin from its substrates [39] and are mostly associated with the stability and/or degradation of cellular proteins. Although this is the first time *USP7, USP15,* and *USP34* have been detected in the cleavage stage mammalian embryos, their high abundance and documented functions suggest that USPs may play an important role in initial development, eg in stabilization against oncogenic insults (USP7 [40]), or in regulation of signalling pathways (USP15 in TGF-β-SMAD pathway [41] [42], and USP34 in in Wnt/β-catenin pathway [43]).

#### RNA splicing

Almost all primary transcripts undergo several splicing events; alternative splicing is a major factor in generating proteomic diversity especially during the early embryonic development. The spliceosome is a highly critical and highly complex piece of cellular machinery [44].

Both 2-cell only and 2-cell enriched transcripts identified by RNA-seq analysis contained several novel candidates involved in RNA splicing. They may play a key role in the regulation of pre-mRNA usage during EGA. One of them is Peter Pan homolog (*PPAN*) which is essential for ribosome biogenesis, and was previously found to be maternally expressed and expressed during early eye and cranial neural crest development in *Xenopus* [45]. Our RNA-seq data represent direct evidence for *PPAN* presence in mammalian development, however, its functional role during initial development remains to be validated. The other candidate is synaptotagmin binding-cytoplasmic RNA interacting protein (*SYNCRIP*) which prevents premature RNA degradation by preferential binding of polyA RNAs and inhibiting deadenylation [46]–[48]. Similarly, splicing factor 3b (*SF3b*) essential for accurate excision of introns from pre-mRNA [49] prevents premature pre-mRNA splicing by keeping the pre-mRNA inactive until the proper spliceosomal conformation is achieved [50].

Interestingly, most of the transcripts identified as enriched before the EGA were functioning in the cytoplasm; PPAN in ribosome biogenesis [51], *SF3b* as integral component of small nuclear ribonucleoprotein (snRNP) assembled in the cytoplasm and then transported to the nucleus to bind pre-mRNA [49], and *SYNCRIP* localized to the cytoplasm [46]. Similarly, other RNA splicing components enriched at the 2-cell stage either function in the cytoplasm; e.g. *SFSWAP* [52], *SMN* 1 and 2, [53], or their activity depends on shuttling between cytoplasm and nucleoplasm, e.g. *PAPOLA* [54], *CDC5L* [55]; *ZRANB2* [56], *HNRNPA3* [57]. Most of the 4-cell stage enriched transcripts, on the contrary, can be directly linked to the activity of RNA polymerase II in the nucleus; e.g. *PRPF19* [58], *ZCCHC8* [59], *KHSRP* [60], *PRPF31* [61], *GTF2F1* [62], *SREK1* [63], *TRA2* [64] or to nuclear import of snRNP (e.g., *snurportin*). Snurportin is a nuclear transport adaptor protein binding to nucleoporins in the nuclear membrane and enabling regulated transport of mature snRNP particles from the cytoplasm to nucleus [65], [66]. Therefore, snurportin may not only be required for delivery of splicing machinery to the newly synthetized RNAs, it may also regulate the onset of EGA indirectly.

In summary, despite the fact that similar processes are taking place before and after EGA, involvement of factors from separate metabolic pathways strongly suggests requirement for a complete metabolic switch. As shown in the example of time-dependent usage of the ubiquitin machinery and the spatial-regulation of the RNA splicing machinery, characterization of this metabolic switch may have significant impact on our understanding of regulation of reprogramming after fertilization and during reprogramming of differentiated cells (eg iPS derivation).

### *In vitro* Culture Conditions Alter RNA Profiles at the Time of EGA

We next examined the differences in RNA profiles between *in vivo*-developed and *in vitro*-produced embryos. For this purpose, the standard method for *in vitro* embryo production was applied, i.e. aspiration of oocytes from gilt ovaries, *in vitro* oocyte maturation followed by *in vitro* fertilization and culture. Gilt ovaries are chosen in the majority of IVF laboratories due to their practical feasibility. Moreover, the quality of cleavage stage embryos is not significantly affected by sexual maturity of the donor [67], and proper sorting of gilt oocytes as used in this study eliminates differences between developmental competence of embryos derived from sow or gilt oocytes [68].

**Figure 4:**
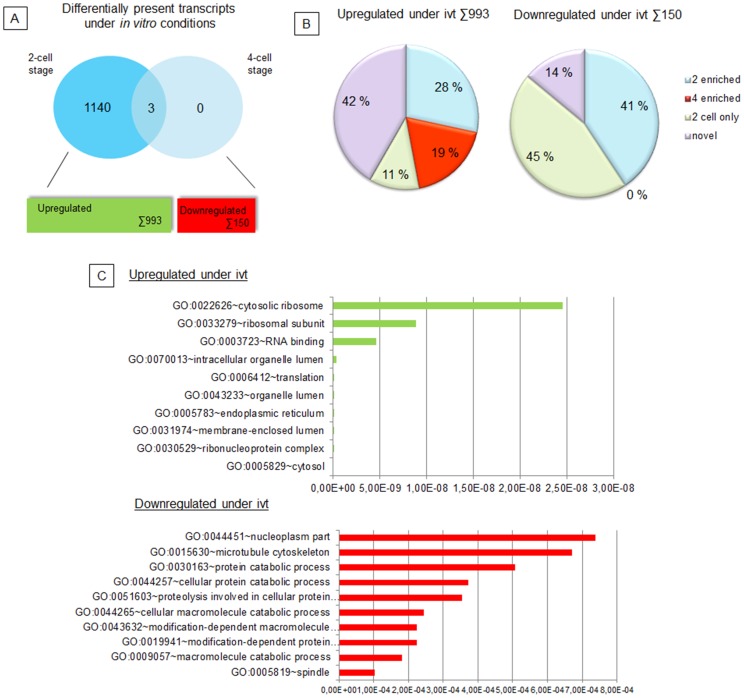
Characterization of genes identified as differentially expressed under *in vitro* conditions. (A) Venn diagram showing distribution of differentially expressed genes according to the cell stage and direction of alteration. (B) Pie graphs showing classification of genes up-regulated (left) and down-regulated (right) under *in vitro* conditions according to their differential enrichment identified by comparison between the 2-cell and the 4-cell stages (2 enriched, 4 enriched, 2 cell only), or not found by this comparison (novel). (C) Graphs showing the top ten significantly enriched GO terms of differentially expressed genes up-regulated (top graph) or down-regulated (below) under *in vitro* conditions.

To compare differential presence of transcripts between 2-cell and 4-cell stage embryos, a new statistical model containing an interaction term between cell stage and embryo source was applied. For each cell stage, 1 replicate vs. 2 replicates of the two embryo sources were compared (for statistical details see Material S1). A total of 1143 genes with significant differential expression between *in vivo* and *in vitro* embryos were identified at the 2-cell stage; whereas only 3 genes were identified at the 4-cell stage. The 3 differentially expressed genes from 4-cell stage were also identified as significantly different at the 2-cell stage, due to which the focus of the analysis was narrowed to the 2-cell stage. From the 1143 genes differentially expressed at the 2-cell stage, 150 were down-regulated and 993 were up-regulated under *in vitro* conditions (Figure 4A).

The 150 down-regulated genes were assigned 84 significantly enriched GO terms forming 35 clusters (Table S5). Interestingly, between the top scored GO term clusters significantly down-regulated under *in vitro* conditions were clusters relating to *protein catabolic processes* (cluster 1), *spindle* and *cytoskeleton* (clusters 3 and 8), *cell cycle regulation* (clusters 5,6,7) (Figure 4C). From all down-regulated transcripts, 45% were categorized as 2-cell only and 41% as 2-cell enriched by previous comparison between the cell stages (Figure 4B). These data indicate that *in vitro* conditions markedly alter the abundance of RNAs involved in these crucial metabolic processes in embryos prior to EGA. The decrease of these transcripts is most probably caused by insufficient synthesis/storage of transcripts during oocyte maturation *in vitro*. Enhanced degradation and/or usage of these transcripts could also contribute to the observed down-regulation.

The most significantly enriched GO term was *spindle*; this term contains genes involved in regulation of microtubular functions during cell cycle progression and chromatin organization. Previous studies have already described alterations in microtubule dynamics caused by vitrification [69], *in vitro* fertilization [70], nuclear transfer [71], and aging during *in vitro* maturation of porcine oocytes [72], [73]. These findings are largely based on ultrastructural observations and/or expression analysis of selected structural proteins. However, ultrastructure assays only allows for assessment of final spindle structures. In addition, if analysing only expression of selected structural proteins as tubulin and CENPB, their RNA levels may be unaffected by *in vitro* procedures [73]. Hence, genome-wide approach as applied in our study revealed new candidates which may be primarily responsible for the observed deregulation of the microtubular compartment. These may be divided into two groups based on their specificity: i) unspecific factors required for mitotic progress (e.g. mitotic checkpoint proteins *BUB1B* and *MAD2L1*) and ii) specific factors related to spatial and temporal regulation of embryonic developmental program (telomere-associated protein *Rif1*, pathways regulator *TBL1XR1* and *Lats1*).

Kinase Bub1b (budding uninhibited by benzimidazoles 1 homolog 1) was previously indicated as suitable marker for oocyte selection for IVF as its down-regulation correlated with restriction in embryo development [74]. Its inefficient expression leads to suppression of cell proliferation and increases abnormal chromosome numbers (Shi et al., 2011). Similarly, down-regulation of *MAD2L1* (MAD2 mitotic arrest deficient-like 1) *in vitro* can abnormally lengthen embryonic M-phase [75] providing explanation of correlation between cleavage rate and developmental competences of *in vitro* produced embryos.

From embryo-specific factors, RIF1 (RAP1 interacting factor homolog) is a telomere-associated protein highly expressed in primordial germ cells, totipotent and pluripotent embryonic cells and stem cells [76]. RIF1 is physically and functionally related to key proteins involved in DNA replication, checkpoint regulation and response to double-stranded DNA breaks (DSBs) [77]. As Rif1 is a multifunctional regulatory protein its decrease in *in vitro* matured porcine oocytes may markedly impair several levels of metabolism (e.g. cycle progression and chromatin repair) and subsequently constrain chromatin remodelling and delay the onset of EGA. Similarly, down-regulation of TBL1XR1 and LATS1 may explain restricted developmental competence of porcine IVT embryos. TBL1XR1 (transducin(beta)-like 1 X-linked receptor) is required for Wnt-β-catenin-mediated transcription [78], which is pre-setting the expression of developmental genes [79]. Lats1 regulates Hippo signalling pathway during gastrulation [80] and cell lineage specification [81]. Therefore, aberrant expression of maternally inherited, cell fate determining factors may result in an inefficient marking of embryonic developmental programs and contribute to decreased developmental competence of IVF porcine embryos.

From the 993 up-regulated transcripts, DAVID analysis revealed 282 significantly enriched GO terms with top scored GO terms as *cytosol*, *endoplasmatic reticulum, translation,* and *RNA binding/processing* (Figure 4C, Table S6). GO terms involved in ribosomal function and RNA processing were also forming the top scored clusters. Classification of the 993 genes into the catogories identified by comparison between the cell stages showed that 39% is 2-cell enriched and thus it would be difficult to distinguish if these are significantly upregulated by *in vitro* conditions. Interestingly, 42% of transcripts were identified as novel, i.e., not found as significantly enriched at either the 2-cell or the 4-cell stage (Figure 4B, Table S7). Novel transcripts annotation gave similar GO terms and GO term clusters as if analysing all 993 up-regulated trasncripts (Table S7). In general, extensive storage (up-regulation) of transcripts responsible for RNA processing and translation under *in vitro* conditions may indicate their insufficient usage and may results in the lack of proteins/factors required at the time of presumptive EGA and cause developmental delay or arrest of the IVF embryos.

In depth analysis of the novel transcripts up-regulated at 2-cell stage under *in vitro* conditions and assigned the most enriched GO term:*Cytosol* revealed a presence of several eukaryotic translation initiation factors (*EIF*). In *in vivo* conditions, EIFs were not found between the differentially present transcripts indicating a balance between their usage and later synthesis. *In vitro* conditions seem to impair this balance and EIF accumulation may reflect accelarated aging caused by increased chromosome instability [82], a phenomenon often seen in oocytes matured *in vitro*; or G1 arrest and apoptosis during hypoxic cell culture conditions [83] or serum starvation [84], which often accompany suboptimal embryo culture.

The most interesting subgroup of transcripts upregulated under *in vitro* conditions at 2-cell stage included 19% (n = 185) of transcripts, which belonged to the 4-cell enriched category (Table S8). These are functionally linked to *mitosis regulation* (Cluster 1 and 7), *cell death* (Cluster 2) and related processes including the GO terms *Lysosome*, *Peroxisome*, *Protein targeting* and *transport*, clusters 3,4,8, and 9, respectively. Whereas some of the apoptosis related proteins are normally found in cycling cells and activate apoptosis upon stress (e.g. *PDCD5* and *NQO1*), others could have more development regulating functions (e.g. *APAF1* and *DIABLO*).

*PDCD5* (programmed cell death 5) and *NQO1* (NAD(P)H dehydrogenase, quinone 1) enhance the stability of free p53 (also upregulated in IVF embryos) and upon induction of stress initiate apoptosis by releasing p53 from stabilizing complexes [85], [86]. *PDCD5* induces apoptosis upon DNA damage commonly seen in IVF porcine embryos as a consequence of polyspermy and/or aberrant mitotic divisions. *NQO1*, on the other hand, mediates the link between p53 and global metabolic status, and may primarily lead to apoptosis [87] or to cell cycle arrest [88]. Activation of these mechanisms may thus reflect suboptimal *in vitro* culture conditions.

*APAF1* (apoptotic peptidase activating factor 1) is a crucial component of apoptosome [89] and was found to be responsible for programmed cell death starting between embryonic day 7 and 9 in mouse [90], [91]. This finding cannot explain its abundant presence at 2-cell stage. However, *APAF1* is also responsible for apoptosis in oocytes and surrounding granulosa cells in growing follicles [92]–[94]. As the IVF system is based on *in vitro* maturation of oocytes (aspirated from tertiary follicles), one can speculate that some of these oocytes have been already determined to undergo degradation by accumulation of *APAF1*. Consequently, accumulated *APAF1* in embryos produced *in vitro* may express its other function as tumor supressor and leads to cell cycle arrest by activation of checkpoint kinase Chk1 [95] in pre-EGA embryos, thereby contributing to the low cleavage rate of porcine IVF embryos. *DIABLO* has been, as the only one from upregulated apoptotic factors, already assigned a function in preimplantation embryos fragmentation and death [96]. Recently it was also shown that *DIABLO* is released from mitochondria into cytoplasm by binding of linker and core histones to outer mitochondrial membrane [97] what may convey genotoxic signals to mitochondria and promote apoptosis following DNA damage under *in vitro* conditions.

In conclusion, the *in vitro* condition significantly alters the fine tuning of initial embryonic development presumably due to aberrations in the transcriptome of the oocytes established already at aspiration or during *in vitro* maturation. As a consequence, embryos have altered cytoplasmic content of several factors, including: i) enhanced of apoptotic factors directing the embryo towards fragmentation and death; ii) impairment of cell cycle regulation factors and spindle components causing developmental block mediated via mitosis check points; and iii) impairment of transcription regulation factors disabling the proper establishment and reading of embryonic genomic program.

### *In vitro* Conditions causes Variability in Gene Expression between Biological Replicates

**Figure 5:**
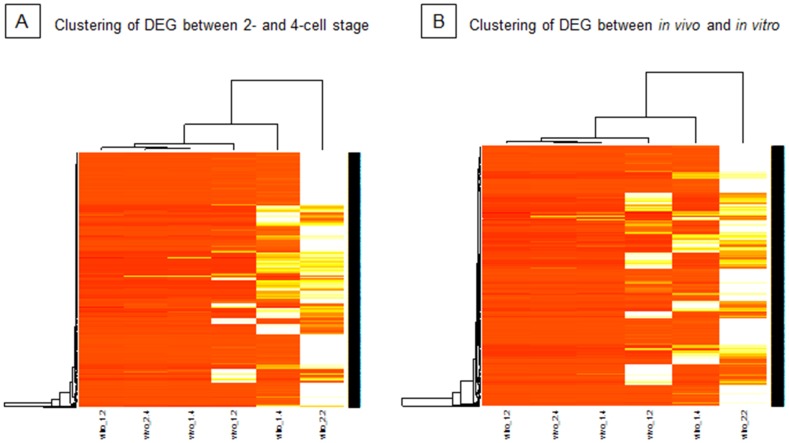
Clustering analysis of differentially expressed genes (DEG). Clustering of genes found as differentially expressed between the 2-cell and the 4-cell stage (A), between *in vivo* developed and *in vitro* produced embryos (B).

Clustering analysis of differentially expressed genes identified by comparison of the 2-cell and the 4-cell stage (Figure 5A) and by comparison of *in vivo*-developed and *in vitro*-produced embryos at 2-cell stage (Figure 5B) showed an interesting relation between the samples. There is very little variation between biological replicates among *in vivo-*developed embryos (vivo_1.4 and vivo_2.4) as these clustered closest. On the other hand, biological replicates of *in vitro-*produced embryos (vitro_1.2 and vitro_2.2) clustered at the highest distance. Thus, *in vitro* conditions cause great variability between the biological replicates. This observation is in agreement with previous studies on *in vitro*-produced porcine embryos assessed at later stages [17].

### RNA Profiles Display Similar Patterns across Mammalian/Vertebrate Embryos

Due to the lack of external RNA profiles of 2-cell porcine embryos, the present results were compared with genome-wide RNA analysis performed on other mammalian species, i.e. the mouse [5] and human [8], [98], or with data derived from studies on porcine MII oocytes and 4-cell stage embryos [99], [100]. These were based on microarray-based transcriptome analysis or on pico-profiling of pre-EGA and post-EGA embryos. Because of the methodological differences and progress in annotation quality between our approach and that used in these citations, we aimed to compare functional groups of transcripts during the EGA. As expected, there was a large overlap between significantly enriched GO terms between the species (Table 5). Transcripts found as enriched before the EGA were annotated GO terms as *mitosis* and *cell cycle regulation, microtubule, protein catabolic processes, RNA splicing and processing,* and *ribosome*, which is in strong correlation with our top 10 enriched GO terms. Moreover, proving the maternally driven development at this stage, GO terms from porcine MII oocyte studies were comparable to those from porcine embryos at the 2-cell stage; i.e. *protein modification and catabolism, cell cycle and mitosis, cytosol, nucleoplasm, microtubule*, etc. The EGA lead to significant enrichment of transcripts assigned GO terms as *RNA/DNA binding*, *transcription regulation, translation initiation, RNA splicing, protein catabolic processes,* as well as *development*.

**Table 5:** Overview of significantly enriched GO terms from analogical stages in external data-sets.

Source	MII oocytes	Prior to the EGA	After EGA onset
Vassena et al., 2011	Nucleic acid metabolism	RNA processing	mRNA transcription and binding
*Homo sapiens*	Protein metabolism	mRNA splicing	Protein targeting
	Protein trafficking	Protein metabolism and modification	Chromatin remodelling
	Cell cycle	Protein biosynthesis	Nucleic acid binding
	Protein metabolism		Transcription regulation
Hamatani et al., 2004	M-phase and mitotic cell cycle	Ribosome/translation factors	RNA binding
*Mus musculus*	Circadian rythm	Proteasome complex	Protein-nucleus import
	Golgi aparatus	RNA binding/processing	Translation initiation
	DNA replication	Protein-nucleus import	Adherent junction
	Intracellular protein transport	Receptor signalling protein	Pyruvate metabolism
Assou et al., 2009	Cytosol	Cell cycle	NA
*Homo sapiens*	Nucleoplasm	Chromatin remodelling	
	Cell cycle	Proteasome	
	Microtubule		
	Protein catabolism		
Whitworth et al., 2005	Mitosis	NA	Microtubule
*Sus scrofa*	Cell cycle		Nucleus
	Microtubule		Development
	Cell adhesion		RNA splicing
	Signal transduction		mRNA binding
Misirlioglu et al., 2006	Transcription regulation	NA	Transcription regulation
*Bos taurus*	DNA methylation		Cell adhesion
	Apoptosis/cell death		Signal transduction
	Protein metabolism		

**Figure 6:**
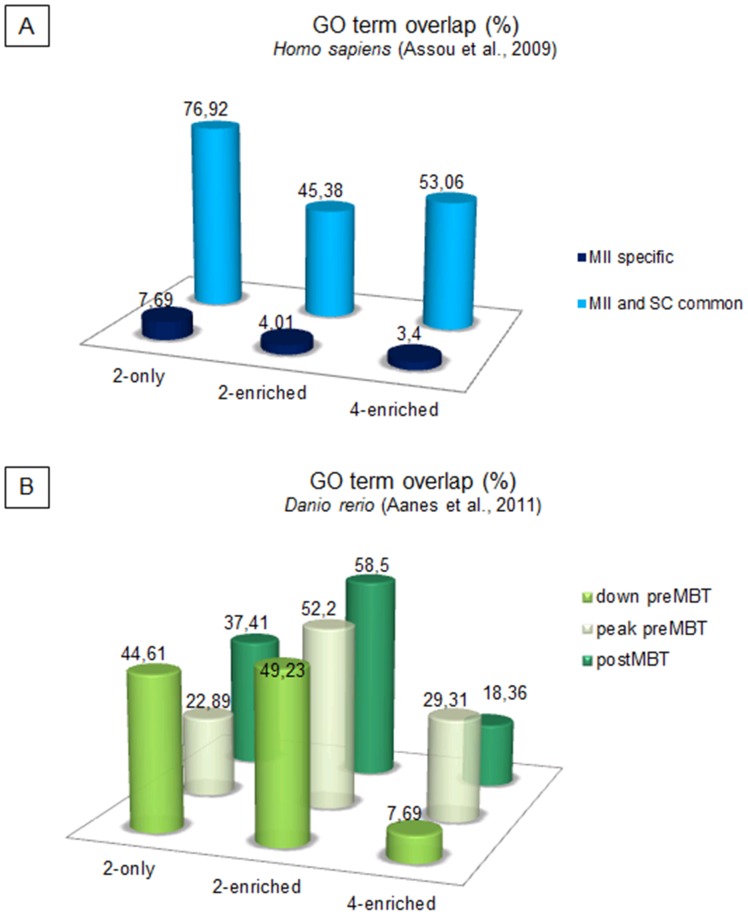
Graphs showing overlap between significantly enriched GO terms in present data and external data-sets. (A) Comparison of significantly enriched GO terms from human MII oocytes and stem cells (Assou et al., 2009) with our data. (B) Comparison of significantly enriched GO terms from zebrafish pre-EGA, EGA and post-EGA embryos (Aanes et al., 2011) with our data.

Interesting insight into reprogramming towards totipotent and early pluripotent state was achieved by comparing the lists of significantly enriched GO terms from human MII oocytes and embryonic stem cells with our data (Table S9) [98]. MII specific transcripts were annotated GO terms, which only in 3.4–7.69% overlap with GO terms found as significant in our study (Figure 6A). On the other hand, looking on GO terms of transcripts which were found as common between MII oocytes and embryonic stem cell lines, gave a large overlap. As expected, the highest similarity (76.92%) was found with the group of 2-cell only transcripts indicating maternally-driven regulation of initial reprogramming towards totipotency. Interestingly, only 45.38% overlap was identified in the group of 2-cell enriched transcripts suggesting the presence of a multitude of specific transcripts required for EGA, which does not occur in dividing stem cells. The 4-cell enriched transcripts overlapped in 53.6% of GO terms signalling the appearance of *de novo* synthetized, embryo-specific transcripts.

Next, we have examined the extent to which the EGA is triggered by the same mechanisms between evolutionary distinct vertebrates. For this purpose, GO term lists from our study were compared with the lists derived from analogical groups of transcripts identified in recent RNAseq study in zebrafish (*Danio rerio*); i.e. transcripts decreasing towards the EGA (down preMBT), transcripts peaking before the EGA (peak MBT), and transcripts increasing after the EGA (postMBT) (MBT, mid-blastula transition) (Table S10) [6]. Unexpectedly, despite the considerable differences in early development between these species (porcine placentation, cleavage rate 24 hrs vs. 15 min in pig and zebrafish, respectively, cell migration and gastrulation occurrence, yolk-dependent nutrition in zebrafish, etc.), transcripts enriched around the time of EGA in zebrafish were assigned ≈ 50% identical GO terms as in pig (Figure 6B, 2-enriched). Moreover, both species showed similar tendencies in GO terms dynamics; e.g. GO terms of transcripts decreasing towards MBT showed highest similarity before the EGA (2-cell only and 2-cell enriched) and only very little overlap after MBT indicating. Similarly, GO terms of transcripts peaking before the MBT in zebrafish showed the highest overlap (52.2%) with GO terms of 2-cell enriched transcripts. Interestingly, only 18.36% of GO terms assigned to postMBT transcripts overlapped with GO terms of the 4-cell enriched transcripts. This may be caused by gaping differences in post-EGA development and thereto related requirement for different transcriptomes. Whereas porcine embryos after the EGA gradually undergo compaction, first lineage segregation, and placentation during several cell divisions, zebrafish at postMBT stage has already undergone substantial cell migration, started gastrulation, and transform within four days into free-feeding larvae.

Overall, our data are in good accordance with previously published, genome-wide profiling data on other species. Moreover, comparison with mouse and human embryos showed striking overlap in functional annotation of transcripts during the EGA.

### Conclusions

Our data constitute the first RNA-seq based transcriptome characterization of porcine embryos at the time of EGA, a critical turn-point in development. The data also constitute one of the few published datasets describing RNA profile changes taking place at the maternal-to-embryonic transition in any species. Particularly, looking at functional groups of transcripts by GO analysis instead of analysing selected transcripts of interest represents an outstanding way of searching for novel and species-universal regulators of initial embryonic development. That, in connection with extensive data mining, may save a lot of time and costs in further research. Moreover, as shown in an example of RNA splicing and protein catabolism, the metabolic switch seems to play a crucial role in transition from highly specialized, differentiated cellular state (sperm, oocyte) into the totipotent state. Therefore, totipotency, while still being underestimated state of cells, may provide much more information about cell plasticity than pluripotency. In general, our data therefore represent a valuable resource available to the scientific community.

## Supporting Information

Table S1 **Gene lists analysed for differential expression by the 2 statistical models (SP = Standard Poission; OP = Overdispersed Poisson) and their log fold changes between 2- and 4-cell stages.**
(XLSX)

Table S2 **GO terms of genes found as differentially expressed between 2- and 4-cell stages divided into the groups of genes present only at 2-cell stage (2-cell only), enriched at 2-cell stage, enriched at 4-cell, and present only at 4-cell stage (4-cell only).**
(XLSX)

Table S3 **Clusters of significantly enriched GO terms for genes found as differentially expressed between 2- and 4-cell stages divided into the groups of genes present only at 2-cell stage (2-cell only), enriched at 2-cell stage, enriched at 4-cell, and present only at 4-cell stage (4-cell only).**
(XLSX)

Table S4 **Lists of differentially expressed genes annotated the GO terms “RNA splicing” and “Protein catabolic processes”.**
(XLSX)

Table S5 **Lists of differentially expressed genes down-regulated under** ***in vitro*** **conditions, including the GO terms annotation and GO term clustering.**
(XLSX)

Table S6 **Lists of differentially expressed genes up-regulated under** ***in vitro*** **conditions, including the GO terms annotation and GO term clustering.**
(XLSX)

Table S7 **Classification of genes differentially expressed under** ***in vitro*** **conditions into the groups of genes found as differentially expressed between 2- and 4-cell stages. List of novel genes found up-regulated under** ***in vitro*** **conditions, including GO terms and GO term clusters.**
(XLSX)

Table S8 **List of differentially expressed genes up-regulated under** ***in vitro*** **conditions at the 2-cell stage and found as enriched first at 4-cell stage by cell stage comparison, including GO terms annotation and GO terms clustering.**
(XLSX)

Table S9 **Comparison of significantly enriched GO terms from human MII oocytes and stem cells (Assou et al., 2009) with our data.**
(XLSX)

Table S10 **Comparison of significantly enriched GO terms from zebrafish pre-EGA, EGA and post-EGA embryos (Aanes et al., 2011) with our data.**
(XLSX)

Material S1 **Details of statistical evaluation of differentially expressed genes between** ***in vivo*** **and** ***in vitro*** **produced embryos at the 2-cell stage and the 4-cell stage.**
(DOC)
