# Supplementary material for: RNA Profiles of Porcine Embryos during Genome Activation Reveal Complex Metabolic Switch Sensitive to In Vitro Conditions
Source: PLoS One. 2013 Apr 29;8(4):e61547. doi: 10.1371/journal.pone.0061547 (PMC3639270; doi:10.1371/journal.pone.0061547)
Supplement: Material S1 — Details of statistical evaluation of differentially expressed genes between in vivo and in vitro produced embryos at the 2-cell stage and the 4-cell stage. (DOC) [file pone.0061547.s011.doc]

**Supplementary material:** Statistical analysis for comparison of *in vivo* and *in vitro* conditions

To accomplish comparison of *in vivo* and *in vitro* derived embryos from the same cell stage, an overdispersed Poisson model was fit with an interaction term between cell stage and embryo source. Inclusion of the interaction term allows for testing of embryo source differences within each cell stage.

1. **Overdispersed Poisson Model with Interaction**

Let $Y_{ijkg}$denote the digital gene expression (DGE) count for gene$g$in the $i^{th}$cell stage with the $j^{th}$embryo source for the $k^{th}$ biological replicate (where $g$=1,…,G; $i$=1,2; $j$=1,2; $k$=1,…,$n_{ij}$; $n_{11}=n_{22}=1$,$n_{12}=n_{21}=2$ ), and let $Y_{ijk.}=\sum_{g} Y_{ijkg}$denote the total number of mapped reads in cell stage $i$ with embryo source $j$for biological replicate$k$. Throughout the analysis, we assume that the proportion of all sequencing reads attributable to gene$g$in cell stage $i$with embryo source $j$for biological replicate$k$ (i.e., $\frac{Y_{ijkg}}{Y_{ijk.}}$) is equivalent to the total number of transcripts in the corresponding sample at the time the mRNA was measured. A Poisson rate model is employed for these data, as this type of model has been effectively used in many RNA-seq applications (Marioni et al., 2008; Auer and Doerge, 2011).

Specifically, to allow for cases where extra-Poisson variation is present, as is typical in RNA-seq data, we fit the following Poisson model that allows overdispersion:

$\pi_{ijkg}^{*}=\upsilon_{ijkg}\exp(\mu_{g}+C_{ig}+E_{jg}+CE_{ijg})$ (1)

where$\lambda_{ijkg}^{*}=\pi_{ijkg}^{*}Y_{ijk.}$ and $\upsilon_{ijkg}$is a random variable with $E(\upsilon_{ijkg})=1$ and . The C, E, and CE terms represent the cell type, embryo source, and interaction effects, respectively. The $\mu$ term represents the overall expression level for each gene across all the samples. A quasi-likelihood approach may be used to obtain estimates of the dispersion parameter ($\varphi_{g}$) for each of the$g$genes:

$\hat{\varphi}_{g}=\frac{X^{2}}{N-p}$ (2)

where $X^{2}=\sum_{i,j,k} \frac{(Y_{ijkg}-\hat{\lambda}_{ijkg})^{2}}{\hat{\lambda}_{ijkg}}$is the Pearson statistic, $N$is the sample size (here, $N=6$), and $p$is the number of parameters (here, (2-1)+(2-1)+(2-1)*(2-1)+1=4). If $\hat{\varphi}_{g}$is much greater than one, then there is evidence for overdispersion. The likelihood ratio test statistic is:

$D_{g}^{2*}=-2\frac{l(\hat{\mathbf{\lambda}}_{0g})-l(\hat{\mathbf{\lambda}}_{Ag})}{\hat{\varphi}_{g}}\sim F_{df,N-p} under H_{0g}$. (3)

Where $df$=1 and $N-p=6-4=2$ for these data.

**2. Pairwise Comparisons**

The main comparisons of interest for this model are to test for differences between embryo sources within the 2-cell stage and 4-cell stage individually. This involves testing a specific set of pairwise comparisons, between embryo stage *j* and *j’* within a given cell stage (*i*). The hypotheses of interest for each gene $g$ are:

$H_{0g}: \pi_{ijg}=\pi_{ij'g}$ vs. $H_{Ag}: \pi_{ijg}\neq\pi_{ij'g}$

The test statistic for this set of hypotheses is based on the statistic $W_{g}=\hat{\pi}_{ijg}-\hat{\pi}_{ij'g}$. The variance of $W_{g}$(Gu et al., 2008) for these hypotheses can be written as:

.

This may be estimated by:

Where $\hat{\pi}_{ijg}$is the MLE of $\pi_{ijg}$from the model in equation 1 and is the estimated dispersion parameter for gene g. Thus, the test statistic may be written as:

$\tilde{W}_{g}=\frac{W_{g}}{s_{W_{g}}}$.

Under the null hypothesis, $\tilde{W}_{g}$is asymptotically distributed as a t(N-p) where N=6 and p=4. To correct for multiple testing, the False Discovery Rate (FDR) was controlled using the Benjamini-Hochberg (BH; Benjamini and Hochberg, 1995) method at 5% for each cell stage. Note that the FDR is controlled separately for each cell stage, rather than jointly, to maintain statistical power (Efron, 2008).


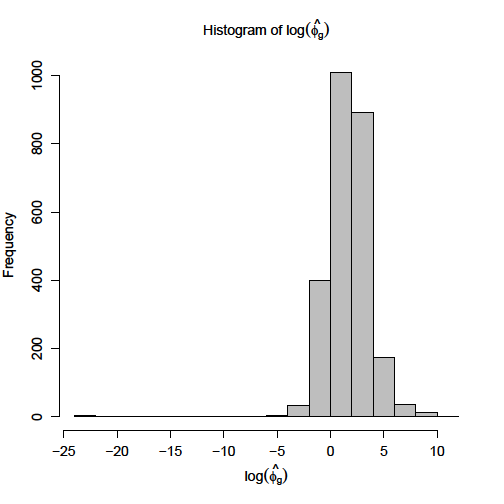


**Figure 1.** Histogram of$\log(\hat{\varphi}_{g})$, the log-transformed estimated dispersion parameters.

**3.** **Results**

The overdispersed Poisson model with interaction (Equation 1) was fit for each of the$g$genes. Genes with expected cell counts (based on the MLEs $\hat{\lambda}_{ijkg}$) for the full model that are larger than or equal to 5 in at least two samples were retained for the analysis, and those not meeting this criterion were excluded in order to maintain the asymptotic behavior of the likelihood ratio test (Cochran, 1952). This leaves a total of 2566 genes for the analysis. A histogram of the log-transformed estimated dispersion parameters is given in Figure 1. We see from this figure that many genes do exhibit overdispersion.

The results of the pairwise comparison tests are displayed in table below. Note that 1143 genes with significant differential expression between the *in vivo* and *in vitro* embryos were identified at the 2-cell stage; whereas only 3 genes were identified at the 4-cell stage. The 3 differentially expressed genes at the 4-cell stage were also identified as significant at the 2-cell stage.

| **Test** | **Model** | **#DE** | **%DE** |
| --- | --- | --- | --- |
| Cell Stage 2*: In vivo* vs. *In vitro* | Overdispersed Poisson with Interaction | 1143 | 44.5% |
| Cell Stage 4: *In vivo* vs. *In vitro* | Overdispersed Poisson with Interaction | 3 | 0.1% |

**4. References**

Auer, P.L. and R.W. Doerge (2011). A Two-Stage Poisson Model for Testing RNA-Seq Data. *Statistical Applications in Genetics and Molecular Biology, 10(1)*, Article 26.

Benjamini, Y. and Y. Hochberg (1995). Controlling the False Discovery Rate: A practical and powerful approach to multiple testing. *Journal of the Royal Statistical Society, Series B 57*, 289-300.

Cochran, W. G. (1952). The $\chi^{2}$goodness-of-fit test. *The Annals of Mathematical Statistics 23*, 315-345.

Efron, B. (2008). Simultaneous inference: When should hypothesis testing problems be combined? *Annals of Applied Statistics* 2: 197-298.

Gu, K., H.K.T. Ng, M.L. Tang, and W.R. Schucany (2008). Testing the ratio of two Poisson rates. *Biometrical Journal* 50: 283-298.

Marioni, J. C., C. E. Mason, S. M. Mane, M. Stephens, and Y. Gilad (2008). RNA-Seq: An assessment of technical reproducibility and comparison with gene expression arrays. *Genome Research 18(9)*: 1509-1517.
